# A systematic approach to evaluate practice-based process- and outcome data applied to the treatment of neovascular age-related macular degeneration

**DOI:** 10.1186/s12886-020-1303-y

**Published:** 2020-01-09

**Authors:** Margriet I. van der Reis, M. Elshout, Tos T. J. M. Berendschot, Yvonne de Jong-Hesse, Carroll A. B. Webers, Jan S. A. G. Schouten

**Affiliations:** 1 Maastricht University Medical Center, University Eye Clinic Maastricht, PO Box 5800, 6202 AZ Maastricht, The Netherlands; 2 Department of Ophthalmology, VU University Medical Center, Amsterdam, The Netherlands; 3 Department of Ophthalmology, Canisius Wilhelmina Ziekenhuis, Nijmegen, The Netherlands

**Keywords:** Age-related macular degeneration; outcome study; visual acuity, Anti-VEGF, Ranibizumab, Bevacizumab, Photodynamic therapy

## Abstract

**Background:**

Following the principles of value-based health care, outcomes and processes of daily-practice eye care need to be systematically evaluated. We illustrate an approach that can be used to support data-driven quality improvements. We used patient data regarding the treatment of neovascular age-related macular degeneration (nAMD).

**Methods:**

In a cohort study, we reviewed medical records of patients with nAMD confirmed on fluorescein angiography (FA). Patients were treated with intravitreal injections with bevacizumab; ranibizumab; or photodynamic therapy (PDT). Visual acuity (VA), ophthalmic exam results and treatments were recorded. VA was compared between treatments by linear mixed model. Diagnosis was re-evaluated on the original FAs. Outcome analysis was performed by 1) selecting VA as the relevant outcome parameter; 2) Preventing selection by comparing treatments with historical untreated cohort and cohorts from the literature, 3) correcting for confounding due to lesion type, and 4) identifying relevant process variables that affect the outcome. These were severity of disease at presentation, and doctor- and patient dependent process variables.

**Results:**

In total, 473 eyes were included. At 12 months, change in VA was 0.54, 0.48, 0.09, and 0.07 LogMAR in the no-treatment, photodynamic therapy (PDT), bevacizumab, and ranibizumab groups, respectively. Lesion type on FA differed between groups. Diagnosis of nAMD could not be confirmed in 104 patients. Patient delay, inaccurate diagnosis and treatment intervals may have impacted outcomes.

**Conclusions:**

The effect of PDT was small to absent. Anti-VEGFs were effective and appeared as effective as in RCTs. Correct selection of a comparator cohort and addressing confounding, including confounding by indication and effect modification, are needed to achieve valid results and interpretation. Patient delay, diagnosis accuracy, indication for and application of treatment can potentially be improved to improve treatment outcomes. In a value-based care perspective, systematic evaluation of diagnostic accuracy, treatment indication, protocols, and outcomes of new interventions is needed at an early stage to improve outcomes.

## Background

Ophthalmology, just as health care systems in general, is moving towards more transparency, accountability, and value-based principles of financing and improving outcomes. Evaluation of the quality of care, based on process data and outcome data is becoming more important. National registration programs and medical records can supply a wealth of data to enable such evaluation. Ophthalmologists can improve their practice by using such systems to analyze and interpret their processes and outcomes.

Ophthalmologists, however, may not be familiar with the data analysis involved. Furthermore, the epidemiological or statistical background, or the interpretation of the results can be challenges. We propose a systematic approach to analyzing medical record data. We show how to interpret results, and to decide whether outcomes can be improved and which variables in the clinical process have affected the outcome.

As an example, we used data regarding interventions for neovascular age-related macular degeneration (nAMD) that have been introduced in the past two decades. Photodynamic therapy (PDT) with verteporfin (VISUdyne®, Novartis) and the anti-vascular endothelial growth factors (anti-VEGF) ranibizumab (Lucentis®, Novartis) and bevacizumab (Avastin®, Roche).

## Methods

We performed a multicenter, retrospective, comparative study, at two tertiary centers and two secondary eye centers in the Netherlands. The Maastricht University Medical Center institutional review board and ethics committee approved the study. The board and committee agreed that permission for this project was not necessary, in accordance with Dutch law. Coordinators at each clinical site approved the study protocol and gave consent to access clinical records.

We selected consecutive patients diagnosed with nAMD treated with PDT, bevacizumab or ranibizumab between 16 July, 2009 and 31 July, 2011. We selected consecutive non-treated patients diagnosed between 1992, and December 31, 1997. During this period, patients with CNV usually received no treatment. Laser photocoagulation was applied for extrafoveal CNV in some instances.

We applied as an inclusion criterion, evidence of subfoveal CNV secondary to nAMD, identified on FA. Further, patients needed to have received at least one treatment with PDT, bevacizumab or ranibizumab for inclusion in a treatment cohort. Exclusion criteria were: participation in an ophthalmological clinical trial, incomplete medical records; no available FA, no record of subfoveal CNV due to nAMD; inappropriate diagnosis on FA, e.g. CNV due to myopia or serous chorioretinopathy. We selected one study eye per patient based on the earliest diagnosis if both eyes of were eligible.

A team of four retina specialists re-evaluated the original FAs: E.C. La Heij, F. Lion, F. Hendrikse and J.S. Schouten. For each FA, two specialists assessed eligibility for inclusion, and the pattern and size of the lesion and its components. CNV components (occult or classic), and non-CNV components of the lesion (thick blood, elevated blocked fluorescence, and serous detachments of the retinal pigment epithelium) were assessed. Definitions of lesion components and location were applied as presented in the 1991 publication by the macular photocoagulation study (MPS) group [1]. Component size and lesion size were measured in disc areas using a template similar to that used in the MPS. Disagreement between assessments was resolved by consensus.

As per the treatment standards, patients with evidence of minimally or predominantly classic lesions, would be treated with PDT. They were treated according to the TAP protocol [2], with follow-up 3 months after treatment. There was a maximum of four PDT treatments per year. Patients treated with ranibizumab or bevacizumab were initially treated with one to three monthly injections. Retreatment criteria varied, partly reflecting early-day treatment strategies. Retreatment was based on the detection of leakage on FA, fluid on optical coherence tomography (OCT), macular hemorrhage, vision loss, or a combination of these criteria. Follow-up intervals ranged from 1 to 3 months.

We collected patient characteristics and general medical and ophthalmic history. For each medical visit, VA, ophthalmic exam and imaging findings, and intervention characteristics were recorded. If patients switched to another treatment, data from beyond the change of treatment were not included. Data was integrated in a secured database (Microsoft Office Access Edition 2003).

We clustered VA results from 2 to 4, 6–8, 10–12, and 22–24 months. The date of first intervention, or the date of diagnosis (in non-treated patients) counted as t = 0. Snellen VA or Early Treatment Diabetic Retinopathy Study (ETDRS) VA was converted to logarithm of the minimal angle of resolution (LogMAR). We assessed for group differences in categorical and continuous variables, using Pearson’s chi-square test and ANOVA, respectively. We used linear mixed models to calculate and compare VA change from baseline. Study group and follow-up time were factors in the models. Age, gender, baseline VA, lesion type, presence of blood, lesion size, and cataract surgery were covariates. We used the statistically significant contributing covariates in the final model. Analyses were performed using SPSS Statistics (IBM, Chicago, IL).

We systematically assessed the processes and outcomes of treatment. We selected VA as the relevant outcome parameter. We compared VA in each treated group with VA in the no-treatment cohort, and the other treated groups. We also compared outcomes with those in randomized clinical trials (RCT). We corrected for confounding variables. Further, we identified relevant patient characteristics and process variables that affect the outcomes.

## Results

**Fig. 1:**
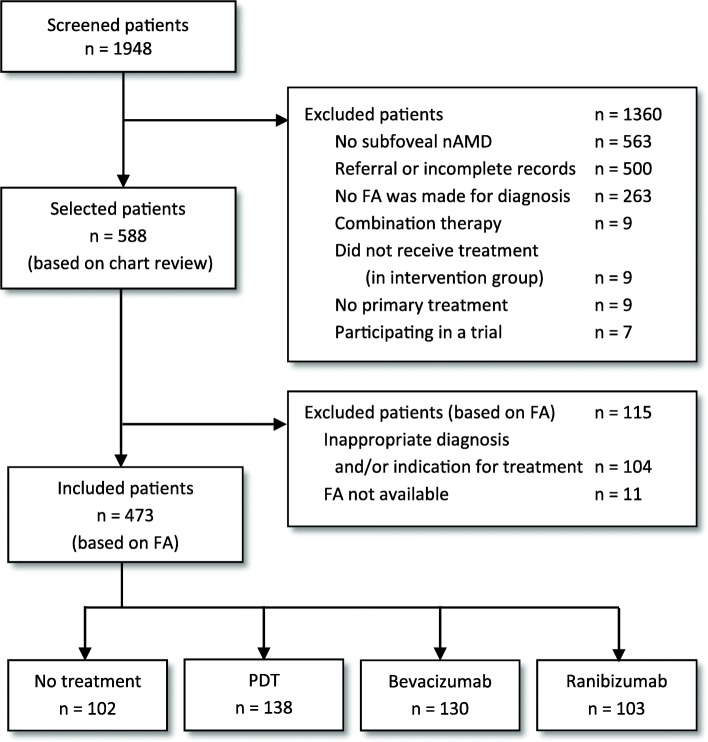
Flow Chart of the Selection Process of Patients*. nAMD*, neovascular age-related macular degeneration; *FA*, fluorescein angiography; *n,* number of patients; *PDT*, Verteporfin photodynamic therapy

Figure 1 shows the process of patient selection. A total of 473 patients (473 eyes) were included. Table 1 presents the baseline characteristics. Mean age was 78.4 years ±7.3 (standard deviation), mean follow-up was 3.2 ± 2.7 years. Groups differed statistically significant in terms of age, lesion type, presence of blood, and CNV classification in the fellow eye. The mean number of interventions was 1.9 (range 1–6), 3.7 (1–9) and 4.3 (1–10) in 1 year for PDT, bevacizumab and ranibizumab, respectively. A switch to another intervention occurred in 28 (20%), 17 (13%) and 33 (32%) patients in the PDT, bevacizumab and ranibizumab groups, respectively. Data from beyond the treatment switch were not included. Forty-one patients in the no-treatment group received photocoagulation. Four PDT patients had previously received photocoagulation (juxtafoveal and extrafoveal). In total, four, five, nine and eleven patients had received cataract surgery during the follow-up in the no-treatment group, PDT, ranibizumab and bevacizumab groups, respectively.

**Table 1:** Baseline characteristics by study group of patients with subfoveal CNV due to age-related macular degeneration

Baseline Characteristics	No Treatment (*N* = 102)	PDT (*N* = 138)	Bevacizumab (*N* = 130)	Ranibizumab (*N* = 103)	*P*-value
Female (%)	51 (50)	79 (57)	77 (59)	54 (52)	0.5
Mean age ± SD	77.7 ± 7.8	76.9 ± 7.5	78.6 ± 6.9	80.8 ± 6.3	< 0.0001
Angiographic lesion subtype^a^					< 0.0001
Predominantly classic (%)	40 (39)	82 (59)	33 (25)	49 (48)	
Minimally classic (%)	11 (11)	16 (12)	24 (19)	10 (10)	
Occult with no classic (%)	46 (45)	36 (26)	59 (45)	36 (35)	
Cannot classify: obscuring lesion (%)	5 (5)	4 (3)	14 (11)	6 (6)	
Cannot classify: photo quality (%)	–	–	–	2 (2)	
Angiographic non-CNV components of lesion^a^					
Elevated blocked hypofluorescence	1 (1)	2 (1)	1 (1)	–	0.7
Blood	36 (35)	74 (54)	58 (45)	54 (52)	*0.02*
PED	6 (6)	8 (6)	15 (12)	6 (6)	0.2
Fibrous tissue	–	–	1 (1)	–	0.5
Angiographic measurements					
Size CNV (disc diameters) ± SD	1.3 ± 0.8	1.4 ± 0.8	1.2 ± 0.8	1.2 ± 0.7	0.3
Missing size CNV	2	1	–	–	
Size of lesion (disc diameters) ± SD	1.8 ± 0.8	1.8 ± 0.8	2.0 ± 0.8	1.9 ± 0.8	0.3
Angiographic classification of fellow eye^b^					< 0.0001
No nAMD (%)	11 (11)	22 (16)	26 (20)	23 (22)	
Early or intermediate nAMD (%)	40 (39)	41 (30)	51 (39)	30 (29)	
Advanced nAMD (%)	49 (48)	64 (46)	51 (39)	35 (34)	
Cannot classify: photo quality (%)	2 (2)	11 (8)	2 (2)	15 (15)	
Visual acuity					
Study eye LogMAR ± SD	0.82 ± 0.49	0.73 ± 0.37	0.73 ± 0.41	0.77 ± 0.48	0.3
Fellow eye LogMAR ± SD	0.67 ± 0.73	0.64 ± 0.75	0.70 ± 0.82	0.64 ± 0.76	0.9
Best eye LogMAR ± SD	0.38 ± 0.33	0.36 ± 0.35	0.35 ± 0.32	0.38 ± 0.40	0.9

*nAMD* Neovascular age-related macular degeneration, *CNV* Choroidal neovascularization, *LogMAR* Logarithm of the minimal angle of resolution, *MPS* Macular photocoagulation study, *N* Number of patients, *PED* Pigment epithelial detachment, *PDT* Verteporfin photodynamic therapy, *SD* Standard deviation

^a^ Definitions based on classification of the Macular Photocoagulation Study (MPS) [1]

^b^ Definitions based on classification of the Age-Related Eye Disease Study Research Group (AREDS) study [3]

### Outcomes

Change in VA in the treated cohorts is shown in Table 2. Visual acuity improved in eyes treated with the anti-VEGFs ranibizumab and bevacizumab in the first months, to slightly decline in long-term follow-up.

**Table 2:** Regression coefficients of the mean changes in visual acuity by study group

Follow-up (months)	Change in VA (LogMAR)						
	No Treatment^a^	PDT (*p*-value)		Bevacizumab (*p*-value)		Ranibizumab (*p*-value)	
2–4	0.27	0.18	(0.2)	−0.01	(< 0.0001)	−0.12	(< 0.0001)
6–8	0.38	0.34	(0.6)	0.002	(< 0.0001)	−0.10	(< 0.0001)
10–12	0.54	0.48	(0.5)	0.09	(< 0.0001)	0.07	(< 0.0001)
22–24	0.69	0.61	(0.5)	0.12	(< 0.0001)	0.13	(< 0.0001)

Note: a positive LogMAR change indicates a decline in visual acuity

*LogMAR* Logarithm of the minimal angle of resolution, *PDT* Verteporfin photodynamic therapy, *VA* Visual acuity

^a^ Reference group

### Comparing outcomes

We compared outcomes in treated groups with VA in the no-treatment group. Table 2 shows VA in non-treated eyes declining over time. We noted no difference between PDT and no treatment. There was a benefit of > 0.4 LogMAR in the anti-VEGFs over no treatment (at 6 to 24 months). We also compared the outcomes to those in RCTs and other daily-practice outcome studies, shown in Table 3.

**Table 3:** Visual acuity in patients with neovascular age-related macular degeneration without treatment in daily practice, or after PDT, bevacizumab or ranibizumab in daily practice and in patients from clinical trials

Regimen	VA Change (LogMAR) at 12 Months				Literature Reference
	From Baseline		Versus Control Group		
	This Study	Literature	This Study^a^	Literature	
No Treatment	0.54	0.2 to 0.4	–	–	[2, 4–6]
PDT	0.48	0.2 to 0.3	−0.06	−0.14	[2, 5–7]
Bevacizumab	0.09	− 0.1 to −0.16	−0.45	−0.33^b^	[8–10]
Ranibizumab	0.07	−0.1 to −0.2	−0.47	−0.35 to −0.41	[4, 7, 8, 10]
PIER study		0.04			[11]
SAILOR study		−0.04			[12]
Anti-VEGF outcome studies		−0.1 to −0.02			[13–26]

Note: a negative LogMAR change indicates an improvement in visual acuity

*LogMAR* Logarithm of the minimal angle of resolution, *PDT* Verteporfin photodynamic therapy, *VA* Visual acuity

^a^ compared to the no-treatment cohort

^b^ compared to ‘standard care’ in the ABC trial

### Addressing confounding

Table 1 shows a difference in the type of CNV lesions between the PDT and anti-VEGF groups. After adjusting for lesion type as a confounder, there was no significant effect of PDT over no treatment, and anti-VEGFs remained equally effective.

### Patient- and process variables that affect outcomes

Disease severity is prognostic for treatment success. In nAMD, severity can be derived from size of the lesion and the presence of blood, shown in Table 1. Diagnostic accuracy is an important process variable. In re-evaluating FAs, we found that a total of 104 patients had not received an appropriate diagnosis of nAMD, or did not have an appropriate indication for treatment. Examples of inappropriate diagnosis were CNV without other hallmarks of AMD such as drusen (e.g. CNV in myopia) and central serous chorioretinopathy. The number of treatments is another important process variable. The number of initial injections was lower, and the time between injections was longer than in current strategies. This may worsen treatment outcomes.

## Discussion

Analysis of daily-practice data is fundamental in improving the value of care. As put forward by M.E. Porter [27]: “Since value is defined as outcomes relative to costs, it encompasses efficiency. Cost reduction without regard to the outcomes achieved is dangerous and self-defeating, leading to false “savings” and potentially limiting effective care.” We undertook a process and outcome analysis for the daily-practice treatment of nAMD. We show the steps involved in such analysis. First, selecting the appropriate outcome parameter. Second, comparing the outcomes appropriately. Fourth, addressing confounding. Finally, identifying relevant process variables that are suitable for improvement. Improving these would complete a feed-back loop, improving daily practice and the value of care.

### Outcome parameter selection

Relevant outcomes can be measurements, expected to change due to treatment. Visual acuity and quality-of-life are examples in the case of nAMD. For comparison purposes, it is useful if the outcome was used in trials. Intermediate outcomes can also be relevant. These reside in the causal pathway from treatment to outcome. Outcomes can then be improved by improving an intermediate outcome. Examples of intermediate outcomes in the case of nAMD are the presence of CNV on FA, edema on OCT, or the presence of blood on the macula.

We used VA as the relevant outcome. It significantly impacts quality-of-life and guides treatment decisions. It is frequently recorded in medical visits, and allows for comparison with RCT results. OCT was not in clinical use during the follow-up of the no-treatment cohort. FA was not commonly repeated in the time-periods before the introduction of effective treatment, and after OCT was introduced. Quality-of-life was not useful in this retrospective analysis, as it is rarely measured in daily practice. There is a risk of bias that is introduced when converting the clinical measure, Snellen VA, to a scale that is suitable for parametric analysis, such as LogMAR. Poor agreement between Snellen and ETDRS charts has been shown previously [28]. The difference tends to be more pronounced in patients with poor vision.

### Comparing outcomes

Outcomes need to be compared, to identify differences which may indicate opportunities to improve diagnosis and treatment. Relevant references and their outcomes depend on the research question. To assess intervention effect, a reference outcome is needed from an untreated cohort. Disease severity and prognosis without treatment should be similar between the treated and the untreated cohort. Selecting an untreated historical cohort from the same setting, probably reflects the characteristics of treated nAMD patients well. This historical cohort should be selected from a period as recent as possible because, for instance, changes in the general population may affect patient characteristics such as average age.

Non-treated patients were selected from the 1992 to 1997 period. We assumed that this group reflected the treated patients’ disease severity and prognosis (had they not been treated). However, there were differences in age, lesion subtype, presence of blood, and fellow eye status (Table 1). These are prognostic factors for nAMD, and therefore may affect outcomes. Importantly, there was no difference in mean VA between groups. As expected, VA decreased over time in the no-treatment group (Table 2). There was no difference in outcome between PDT and no treatment. There was a marked difference in outcome with the anti-VEGFs. A lower prevalence of classic CNV in the no-treatment group may be an explanation. This lower prevalence may imply that the no-treatment group does not reflect the theoretical PDT groups’ prognosis (without PDT). However, even after adjusting for baseline characteristics such as CNV type and baseline VA, there was no difference in outcome between PDT and no treatment, and the anti-VEGFs still had statistically significant better outcomes compared to the no-treatment group at 10–12 months.

Comparing a treated group with an untreated group from the same time period may seem attractive. However, this introduces a high risk of confounding by indication, inducing interpretation errors. In nAMD, patients with mild disease and good prognosis may be left untreated. Patients with severe disease with poor prognosis, such as severe macular hemorrhage, may also be left untreated. Comparing anti-VEGF-treated patients with a no-treatment group that includes such patients will not produce meaningful results, as disease severity and prognosis without treatment are not comparable. We decided not to compare with an untreated group from the same time period.

Outcomes can also be compared between treatments. An advantage is that the indication for treatment is more likely to be similar. However, in nAMD, PDT and anti-VEGF have different indications. Therefore, we compared the anti-VEGFs and PDT, and adjusted for baseline differences in, for example, CNV type. We found that bevacizumab and ranibizumab had better outcomes. There was no difference in outcome between bevacizumab and ranibizumab (Table 2).

Daily-practice outcomes can be compared to RCT outcomes. A difference in outcome may provide indications for improving clinical practice. There are important differences between clinical practice and RCTs. For instance, VA may not be measured as accurately in daily practice as in RCTs, while such measurements do determine the decision-making by clinicians. In RCTs, patient selection is generally strict. This can yield a population with better established diagnosis, which could lead to a better treatment outcome. Inclusion criteria may select patients that are more sensitive for improvement with a specific intervention, or with a more severe disease status. Follow-up can be more frequent, with better equipment and higher-quality assessment of re-treatment criteria. Compliance-enhancing strategies could have been implemented. To assess for differences in patient populations’ disease status, the untreated daily-practice group can be compared with the RCT’s placebo group (Table 3). The VA change at 12 months in our no-treatment group was worse than in RCT placebo groups. This implies that prognosis was worse in our patients. Next, the outcomes of treatment in daily-practice and in the RCTs can be compared. For PDT, the daily-practice outcome appeared worse than that in RCTs (Table 3). However, offsetting PDT outcomes to the corresponding untreated groups (yielding the ‘effect size’) produces benefits in VA in both daily practice and RCTs. However, outcomes in daily practice were still worse than in RCTs and not statistically different from no treatment. This suggests a need for further assessment of differences in patient and treatment characteristics. This could indicate possibilities to improve patient selection or improve the treatment protocol.

Regarding anti-VEGF treatments, outcomes appeared worse in daily-practice than in RCT treatment arms (Table 3). However, the effect size over the non-treated group is greater in our study than in RCTs. Relevant prognostic factors were taken into account. Thus, effectiveness of anti-VEGF appeared as good as in RCTs. The outcomes of our study align more with the treated patients in the PIER and SAILOR study, which showed a lower gain in VA from baseline compared to earlier RCTs. In these studies, patients received a reduced number of treatments due to extended follow-up intervals of 3 months. A reduced number of injections by itself will not lead to a lower VA if monthly follow-up takes place and injections are given pro re nata based on adequate criteria [8, 29–31]. However, to achieve optimum outcome, 3 months follow-up period is too long [32].

Finally, outcomes in daily practice can also be compared to outcomes of other retrospective outcome studies. Those studies found VA to stabilize or decline after 1 year of anti-VEGF treatment [33–35]. Tao et al. found a VA decline after 2 years [36]. In contrast, a majority of outcome studies found a VA improvement of at least − 0.1 logMAR after 1 year treated with anti-VEGFs (Table 3). A very large outcome study in over 11,000 patients showed VA stabilization over a period of 3 years with a yearly median number of 4–5 injections and 8.2 to 9.2 outpatient visits [13].

### Addressing confounding

In comparing outcomes between cohorts, it is important to consider the cause of the observed difference. For example, is a difference in VA after treatment with PDT or anti-VEGF is really caused by the difference in treatment? To answer this question, confounding variables need to be taken into account. Relevant variables are prognostic factors, or factors that modify the effect of treatment. In comparing PDT and anti-VEGF, we needed to adjust for CNV lesion type, since PDT was not indicated for all lesions, and we observed a difference in lesion types between PDT and anti-VEGF groups (Table 1). Furthermore, lesion type determines the prognosis [37–39]. We found that, after adjusting for lesion type, occult or classic CNV, there was no effect of PDT.

Comparing the effect size of ranibizumab treatment over no-treatment in daily practice to the effect size in an RCT, effect-modifying variables need to be taken into account. Lesion type and size can be effect-modifying variables. The effect size is greater in classic CNV and in small lesions than in occult CNV and in larger lesions [38]. In considering effect modification, a stratified analysis is needed based on varying levels of the effect-modifying variable. This analysis shows whether the effect size differs between different levels of the variable. Lesion type as an effect-modifier was taken into account to assess the difference in outcome between PDT and anti-VEGF.

Confounding by indication is a special case of confounding. In comparing PDT or anti-VEGF-treated patients to untreated patients from the same period, a difference in outcome can be due to confounding by indication, even after taking other confounding variables into account. Ophthalmologists consider patients eligible for treatment based on prognosis or expected treatment effect. Therefore, a priori, the outcome is different between treated and untreated patients from the same period. Comparing two treatments with different selection criteria may introduce similar confounding. In our study, after taking confounding factors (lesion type) into account, there still was a difference in outcome between PDT and anti-VEGFs. To a high degree of validity, anti-VEGFs were more effective in daily practice than PDT, unless the difference is due to confounding variables that were not measured, or are difficult to measure, such as confounding by indication.

### Identifying the factors that affect outcomes

The ultimate goal of outcome- and process-analysis is identifying factors to improve in the process of patient selection, diagnosis and treatment. In the previous sections, we outlined the relevant factors that determine outcomes. We described methods to determine the effectiveness of the treatments, the effect sizes, corrected for confounding, with the major pitfalls. In the following we describe the next step of addressing several process factors that commonly affect the outcome: severity of the disease at first presentation; physician-dependent process variables; and patient-dependent process variables.

The severity of the disease determines the prognosis, the appropriate intervention, and the outcome. We identified several nAMD severity factors: lesion type; lesion size and presence of blood in the lesion (see Table 1). These variables determine the prognosis without treatment, and the effect of treatment with e.g. PDT and anti-VEGF [37, 38]. Earlier referral to an ophthalmologist may lead to a lower severity of the disease at baseline. Literature indicates that delay access to a general practitioner or late referral to an ophthalmologist occurs and influences the outcome of nAMD treatment [40]. Earlier access to an ophthalmologist is a focus for improvement if the outcome of nAMD is to be improved.

Assessing physician-dependent process-variables includes assessing whether the diagnosis and the indication for treatment were appropriate, and whether the treatment or intervention was correctly timed and applied. We re-assessed the diagnosis and the indication for treatment (subfoveal CNV due to nAMD) in our study. They were not correct in 104 patients. Classification of CNV type or assessment of retreatment criteria tends to differ between ophthalmologists [41, 42]. Treatment is unlikely to be effective without the presence of CNV. In addition, literature indicates that the timing of the intervention may impact the outcome [43, 44]. Improving diagnosis and indication appropriateness will improve outcomes. Measuring the incidence of diagnostic error in everyday practice is an essential requirement of a comprehensive quality management program [45]. Having diagnostic confirmation before commencing treatment is paramount. Treating a patient for a disease based on inaccurate diagnosis leads to unpredictable results and delays proper treatment.

Sometimes, treatment schedules in clinical practice may not be according to the “state-of-the-art.” In this regard, improving care requires a definition of the state-of-the art by guidelines or clinical trials. In our study, PDT was given according to the TAP protocol with a follow-up period of 3 months and a maximum of four PDT treatments per year [2]. Early after the introduction of anti-VEGF treatments, some repeated treatments with anti-VEGF were applied with longer intervals as compared to early RCTs. In the RCTs, monthly treatments or PRN treatment were applied [4, 7, 11, 46–50]. Using a different treatment protocol may not necessarily worsen outcomes. Ophthalmologists may individualize treatment to optimize outcomes, and they may take into account a strategy preferred by the patient, or may prioritize avoiding adverse events over other potential benefits of treatment. However, we now know that three-month intervals between anti-VEGF injections of bevacizumab as a standard is too long and adversely affects the outcome [11].

Patient-dependent process variables involve the timely reporting of symptoms by the patient, and adherence to prescribed medication, to follow-up appointments and to life-style advice. We did not specifically address these factors in our study, as it was based on a medical record review. In nAMD, timely reporting of symptom recurrence and timely follow-up are known to be important patient related-process variables that impact the outcome [43, 51, 52].

## Conclusions

With this article, we contribute to the opportunities for ophthalmologists to participate in data analysis and discussions in the era of value-based health care. These discussions are becoming increasingly important since value-based health care is seen as a “breakthrough that will change the face of medicine, the goal of which is to lower health care costs and improve quality and outcomes” [53].

As supported by this study, in the case of nAMD, preventing patient and doctor delay, improving the appropriateness of diagnosis and indication for treatment, considering anti-VEGF over PDT, and addressing the frequency of follow-up and frequency, anti-VEGFs will improve the value for patients.

## Data Availability

The datasets generated and analysed during the current study are not publicly available in order to protect patient identity and confidentiality, but are available from the corresponding author on reasonable request.
